# Assessment of the Chemosensitizing Activity of TAT-RasGAP_317-326_ in Childhood Cancers

**DOI:** 10.1371/journal.pone.0120487

**Published:** 2015-03-31

**Authors:** Nadja Chevalier, Nicole Gross, Christian Widmann

**Affiliations:** 1 Department of Physiology, University of Lausanne, Lausanne, Switzerland; 2 Paediatric Oncology Research Unit, University Hospital Center (CHUV), Lausanne, Switzerland; Institute of Hepatology - Birkbeck, University of London, UNITED KINGDOM

## Abstract

Although current anti-cancer protocols are reasonably effective, treatment-associated long-term side effects, induced by lack of specificity of the anti-cancer procedures, remain a challenging problem in pediatric oncology. TAT-RasGAP_317-326_ is a RasGAP-derived cell-permeable peptide that acts as a sensitizer to various anti-cancer treatments in adult tumor cells. In the present study, we assessed the effect of TAT-RasGAP_317-326_ in several childhood cancer cell lines. The RasGAP-derived peptide-induced cell death was analyzed in several neuroblastoma, Ewing sarcoma and leukemia cell lines (as well as in normal lymphocytes). Cell death was evaluated using flow cytometry methods in the absence or in the presence of the peptide in combination with various genotoxins used in the clinics (4-hydroperoxycyclophosphamide, etoposide, vincristine and doxorubicin). All tested pediatric tumors, in response to at least one genotoxin, were sensitized by TAT-RasGAP_317-326_. The RasGAP-derived peptide did not increase cell death of normal lymphocytes, alone or in combination with the majority of the tested chemotherapies. Consequently, TAT-RasGAP_317-326_ may benefit children with tumors by increasing the efficacy of anti-cancer therapies notably by allowing reductions in anti-cancer drug dosage and the associated drug-induced side effects.

## Introduction

Cancer represents the second cause of death in children after accidents in industrialized countries [1, 2]. Our understanding of childhood cancers has benefited from significant advances over the four last decades. Standard treatments to cure pediatric tumor include surgery, radiation therapy and intensive multi-agent chemotherapy such as etoposide, vincristine, doxorubicin, and cyclophosphamide [3].

In developed countries, eighty percent of children who are diagnosed with cancer are expected to survive within 5 years following the treatment. However, most of them will suffer from chronic diseases by 40 years of age [4]. Extended surveillance of pediatric cancer survivors shows a high risk for life-threatening late effects from second malignancies, cardiac conditions and pulmonary diseases [5, 6]. The risk of early mortality is mostly determined by treatment-specific factors such as the cumulative dose of chemotherapy [7]. Therefore, treatment-associated long-term side effects induced by damage to non-tumor cells are a challenging problem and remain largely unresolved. As children are by definition long-term survivors, there is a strong need to develop low-toxicity, better targeted and efficient new therapeutic strategies for all types of childhood cancers [8]. Strategies to circumvent such obstacles include the improvement of anti-cancer drug sensitivity and specificity toward cancer cells [9–11].

In this context, we previously reported that a cell-permeable peptide derived from the p120 RasGAP protein, called TAT-RasGAP_317-326_, is a tumor-sensitizer to various anti-cancer drugs. Indeed, although it does not show any toxicity toward cells on its own, it efficiently and specifically sensitizes adult tumor cells *in vitro* and *in vivo* to various anti-cancer therapies, including chemotherapy [12,13], and photodynamic therapy [14]. Importantly, it displays specificity to cancer cells as it does not sensitize non-tumor cells to genotoxin-induced apoptosis [12, 14]. TAT-RasGAP_317-326_ appears to have additional anti-cancer activities than tumor cell sensitization as it has been recently demonstrated that this peptide can hamper cell migration and invasion *in vitro* [15, 16] and that this activity can inhibit the metastatization process *in vivo* [17]. This indicates that the RasGAP-derived peptide has the ability to act as an anti-metastatic compound on top of its tumor sensitization effects. Recently, it has been shown that the anti-cancer properties of TAT-RasGAP_317-326_ are dependent on two tryptophan residues at position 317 and 319 [16]. However, the mode of action of TAT-RasGAP_317-326_ is not fully characterized. It has been previously shown that this peptide does not favor the death of tumor cells by modulating Ras activity, MAPK signaling pathways, NF-κB transcriptional activity, Akt protein levels and phosphorylation status [18, 19]. Moreover, the Bcl-2 family members, which regulate mitochondrial-dependent cell death, were shown to be individually dispensable for the sensitizing activity of the peptide [20].

The effect of this peptide in childhood cancer is however not known. The molecular biology of pediatric tumors is distinct from cancers in adults in many ways. As the genesis of most childhood cancers seems to come from disruptions of normal early development, they accumulate fewer mutations than adult tumors. On the other hand, it appears that development of pediatric tumors rely heavily on epigenetic modifications [21–23]. In the present study, we have therefore investigated whether TAT-RasGAP_317-326_ was able to render childhood tumors more sensitive to clinically relevant anti-tumor drugs.

## Methods

### Cell lines and culture cells

The CCRF-CEM [24], THP-1 [25] and A673 [26] cell lines were obtained from the American Type Culture Collection (ATCC) (references CRL-8436, TIB-202, CRL-1598 respectively). The LAN-1 [27] and M-07e [28] cell lines were obtained from the Leibniz Institute DSMZ-German Collection of Microorganisms and Cell Cultures (references ACC655, ACC104 respectively). The EW-11 [29], TC252 [30] and NB1-derived [31] cell lines were described earlier. All cell lines were cultured in 5% CO_2_ at 37°C. The neuroblastoma cell lines (LAN-1, NB1-FBS, NB1-FBS-Re) and the EW-11 Ewing sarcoma cell line were grown in Dulbecco’s modified Eagle Medium (DMEM) (Gibco, Paisley, UK) containing 10% fetal bovine serum (FBS) (Gibco). The NB1-NBM neuroblastoma primary tumor cells were maintained in neural basic medium made of DMEM/F12 (Gibco) supplemented with 2% B27 serum-free supplement (Invitrogen, Carlsbad, CA), 20 ng/ml human recombinant basic fibroblast growth factor (bFGF) (Peprotech), and 20 ng/ml human recombinant epidermal growth factor (EGF) (Peprotech). The CCRF-CEM and THP-1 acute leukemia cell lines, and the A673 and TC252 Ewing sarcoma cells, were cultured in RPMI 1640 (Gibco) containing 10% FBS. M07e, an acute myeloid leukemia cell line, was maintained in Minimum Essential Medium alpha (MEMα) (Gibco) supplemented with 10% FBS and 5 ng/ml recombinant human granulocyte macrophage colony-stimulating factor (GM-CSF) (Peprotech). Human peripheral blood lymphocytes (PBLs) were isolated by density centrifugation over a Ficoll-Paque gradient (Lymphoprep; Stemcell Technologies) from buffy coats of healthy human donors, obtained from the state of Vaud blood transfusion service. The donors gave written consent for potential use of their blood for medical research. PBLs were cultured in RPMI 1640 (Gibco) supplemented with 8% of pooled human serum and supplemented with 100 U/ml recombinant human interleukin-2 (Proleukin, Roche Pharma AG).

### Chemicals

The drugs used were vincristine (Sigma-Aldrich, St Louis, USA), etoposide (Sigma-Aldrich), 4-hydroperoxycyclophosphamide (4-HC) (Niomech, Bielefeld, Germany) and doxorubicin (Pfizer AG, Zurich, Switzerland). The staining used to perform flow cytometry were 7-aminoactinomycin D (7-AAD) and Annexin V-FITC (Annexin V-FITC/7-AAD kit, Beckman Coulter, Miami, USA).

### Peptide synthesis

TAT-RasGAP_317-326_ (GRKKRRQRRRGGWMWVTNLRTD), TAT_48-57_ (from now on referred to as TAT) (GRKKRRQRRR), TAT-Scrambled (GRKKRRQRRRGGWLDMTTVNRW) and TAT-Mutated (GRKKRRQRRRGGAMWVTNLRTD) were synthesized at the Department of Biochemistry, University of Lausanne, Switzerland as described previously [12].

### Cytotoxic assay

For cells growing in suspension (THP-1, M07e, CCRF-CEM, and lymphocytes), three hundred thousand cells were seeded in 6-well plates and directly treated with the indicated doses of 4-HC, etoposide, vincristine, or doxorubicin in the presence or in the absence of 10 μM TAT-RasGAP_317-326_, TAT, TAT-Scrambled or TAT-Mutated. For adherent cell lines (NB1-NBM, NB1-FBS, NB1-FBS-Re, LAN-1, EW-11, TC252, and A673), two hundred thousand cells were allowed to adhere for 48 hours in 6-well plates and were then treated like cells growing in suspension. Twenty-four hours after drug incubation, cells were subjected to flow cytometry to evaluate cell death. Briefly, adherent cell lines were detached by trypsin-EDTA (Gibco) and single-cell suspensions were processed and stained with 1 μg/ml 7-AAD and 50 ng/ml Annexin V-FITC in 500 μl binding buffer. A minimum of ten thousand cells were analyzed. The 7-AAD dye-derived signal could not be used when cells were treated with doxorubicin because 7-AAD and doxorubicin have similar fluorescent properties (7-AAD emission wavelength: 525 nm; doxorubicin emission wavelength: 530 nm [32]).

### Statistical analysis

Unless otherwise mentioned, all experiments were derived from three or more independent experiments. The results were expressed as mean ± 95% confidence intervals. Significance was assessed using t-tests performed with Microsoft Excel 2010 followed by Bonferroni corrections (i.e. differences were considered significant when p values < 0.05/n, where n is the number of comparisons made). Asterisks represent statistically significant differences. In the last figure, significance was assessed by one-way ANOVAs followed by Bonferroni (Dunn) t post-hoc tests using the SAS 9.2 software (SAS Institute Inc., Cary, NC, USA).

## Results

In this study, three types of childhood cancers were analyzed: neuroblastoma, leukemia, and Ewing sarcoma. Among childhood malignancies, neuroblastoma is the most frequent extracranial solid cancer. Deaths resulting from neuroblastoma account for 15% of all tumor related death in children [33]. Multidrug resistance, acquired during exposure to chemotherapies, plays a major role in this poor outcome [34, 35]. Leukemia is the most common childhood cancer type and represents one third of all pediatric tumors [36]. Although less prevalent than leukemia, Ewing sarcoma is an aggressive cancer with a poor prognosis and a high rate of relapse with metastatic disease [37]. The drugs employed here are all used in the clinics. Their properties are presented in Table 1.

**Table 1:** Mechanism of action and clinical use of cyclophosphamide, doxorubicin, etoposide and vincristine.

Drugs	Class	Mechanism of action	Clinical use
Cyclophosphamide	Alkylating agent	Interstrand DNA crosslinker	NB, ES, ALL, AML
Doxorubicin	Antracycline	DNA intercalation and inhibition of the progression of topoisomerase II enzymes	NB, ES, ALL, AML
Etoposide	Topoisomerase inhibitor	Single or double strand breaks by trapping topoisomerase II enzymes on DNA	NB, ES, AML
Vincristine	Mitotic inhibitor	Inhibition of assembly of microtubule structures	NB, ES, ALL, AML

Only the tumor types that were analyzed in this study are mentioned here. The listing was based on the protocols of the Children’s Oncology Group and the International Society of the Pediatric Oncology (EURO-E.W.I.N.G. 99; AALL0232; HR-NBL-1.5/SIOPEN). NB, neuroblastoma; ES, Ewing sarcoma; ALL, acute lymphoblastic leukemia; AML: acute myeloid leukemia.

### TAT-RasGAP_317-326_ sensitizes acute lymphoblastic and myeloid leukemia cells to various chemotherapeutic agents without showing any effect toward tumor cells by itself

**Fig 1:**
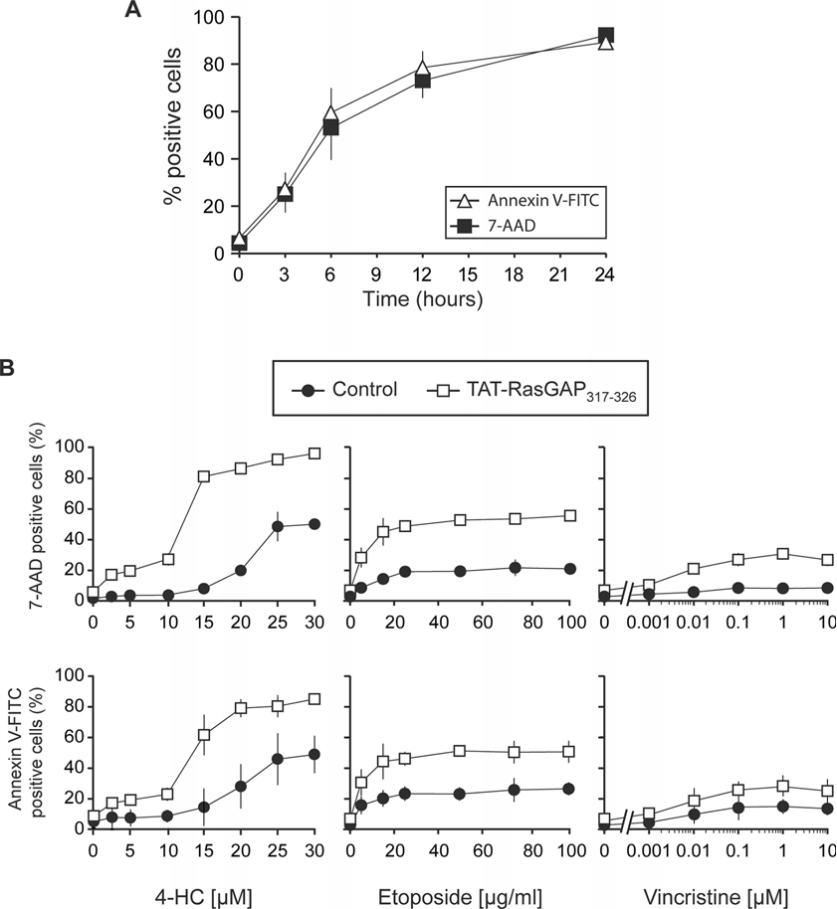
Necrosis-like death induced by 4-HC in CCRF-CEM cells. **A.** Three hundred thousand CCRF-CEM cells were seeded in 6-well plates and directly treated with 50 μM 4-HC. Cell death was evaluated after several time points (0, 3, 6, 12 and 24 hours) using 7-AAD and Annexin V-FITC staining. **B.** CCRF-CEM cells were seeded in 6-well plates and directly treated with the indicated doses of 4-HC, etoposide or vincristine in the presence or in the absence of 10 μM TAT-RasGAP_317-326_. After 24 hours of drug incubation, 7-AAD and Annexin V-FITC staining was performed to evaluate cell death. 4-HC, 4-hydroperoxycyclophosphamide.

**Fig 2:**
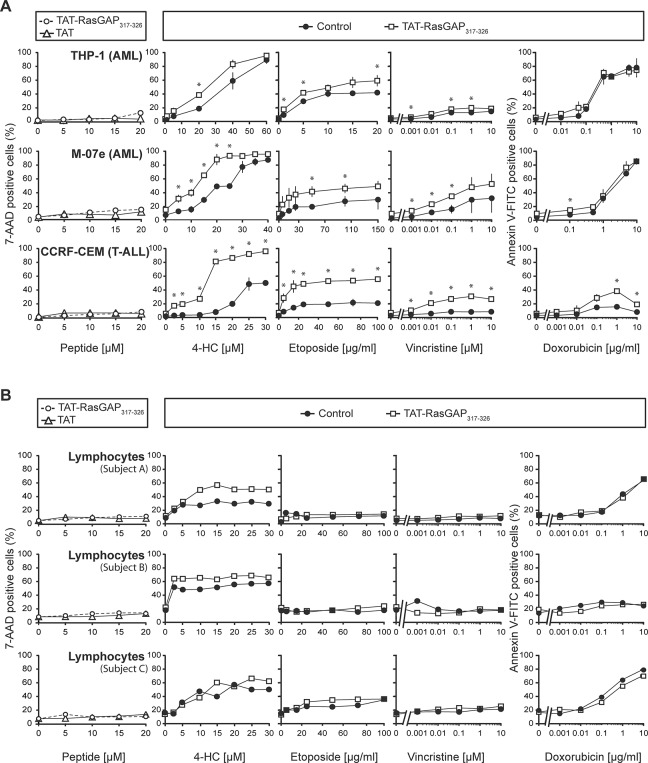
The effect of TAT-RasGAP_317-326_ as a chemosensitizer of leukemia cells and non-tumor lymphocytes. **A.** Two acute myeloid leukemia cell lines (THP-1 and M-07e) and one T acute lymphoblastic leukemia cell line (CCRF-CEM) were seeded in 6-well plates and directly treated with 4-HC, etoposide, vincristine or doxorubicin at the indicated concentrations in the presence or in the absence of 10 μM TAT-RasGAP_317-326_. After 24 hours of drug incubation, 7-AAD or Annexin V-FITC staining was performed to evaluate cell death (last four columns). Alternatively (first column), the cells were treated with increasing concentrations of TAT or TAT-RasGAP_317-326_ alone. After 24 hours, the evaluation of cell death was carried out using 7-AAD staining. **B.** Isolated lymphocytes from three distinct healthy subjects were treated as described in Fig. 2A. The dosages of chemotherapeutic agents used to treat the healthy lymphocytes from the three subjects are similar to those used to treat the T-ALL CCRF-CEM cells. Note that the graphs are derived from single experiments where lymphocytes are immediately used after their isolation (if cultured *in vitro*, they would experience high levels of spontaneous apoptosis that would prevent accurate measurement of anti-cancer drug- and peptide-induced death). T-ALL, T-acute lymphoblastic leukemia; AML, acute myeloid leukemia; 4-HC, 4-hydroperoxycyclophosphamide. * p<0.05 t-test after Bonferroni correction.

To investigate the sensitization effect of TAT-RasGAP_317-326_ in leukemia cells, three different cell lines were subjected to increasing concentrations of cytostatic agents in the absence or in the presence of 10 μM TAT-RasGAP_317-326_. Etoposide, vincristine, doxorubicin, and 4-hydroperoxycyclophosphamide (4-HC), the active form of cyclophosphamide, were the drugs used here and are each currently employed in the clinic. Cell death was assessed using 7-AAD that labels dead cells that have permeabilized plasma membrane and with Annexin V that binds to phosphatidylserine exposed on dead cells. Some of the anti-cancer drugs used here induced a concomitant appearance of the 7-AAD and Annexin V signals (Fig 1A shows the representative case of 4-HC). In other words, as soon as a cell became Annexin V-positive it also picked up the 7-AAD dye. Hence, in our hands, anti-cancer drugs such as 4-HC induced a necrosis-like type of death. In the next figures, the 7-AAD data are reported as we found them to be associated with a lower variance than those obtained with Annexin V staining (Fig 1B). The only exception was when doxorubicin was used. Indeed, this dye fluoresces at similar wavelengths as 7-ADD. In this case therefore, the Annexin V data are presented. These experiments revealed that TAT-RasGAP_317-326_ significantly sensitizes leukemia cells to almost all tested drugs (Fig 2A). However, in some conditions (for example when THP-1 cells are treated with vincristine) tumor cells did not respond well to TAT-RasGAP_317-326_. A limited sensitization effect of the RasGAP-derived peptide is not necessarily a consequence of intrinsic resistance to a drug as the CCRF-CEM cell line, which is vincristine-resistant, was efficiently sensitized by TAT-RasGAP_317-326_. This point is of particular clinical relevance as it indicates that TAT-RasGAP_317-326_ can exert a sensitization effect in chemo-resistant cells.

TAT-RasGAP_317-326_ by itself did not induce cell death in the tested leukemia cell lines, even at a two-fold higher concentration (20 μM) than the one used to induce a sensitization effect (10 μM) (Fig 2A).

### TAT-RasGAP_317-326_ does not display toxicity toward non-tumor lymphocytes from healthy subjects.

It was shown previously using non-tumor immortalized human keratinocytes (HaCaT) and umbilical vascular endothelium (HUV-EC-C) cells that TAT-RasGAP_317-326_, in combination with various genotoxins, did not sensitize non-tumor cells [12]. In the present study, we used isolated lymphocytes from whole blood of healthy patients to investigate the selectivity of the RasGAP-derived peptide toward cancer cells. We used the same dosages of chemotherapeutic agents to treat healthy lymphocytes as those used to treat the CCRF-CEM T-acute lymphoblastic leukemia (T-ALL) cell line (Fig 2A). Fig 2B shows the response of peripheral blood lymphocytes (PBLs) derived from three different healthy subjects (subjects A-C) to the RasGAP-derived peptide in combination or not with the genotoxins used in Fig 2A. The RasGAP-derived peptide by itself did not display any toxicity toward the PBLs. The sensitivity of the non-tumor lymphocytes for the tested chemotherapies alone was similar to the sensitivity of the CCRF-CEM cell line (even slightly increased in the case of doxorubicin for the healthy subjects A and C). The observation that cancer and normal lymphocytes were similarly sensitive to genotoxins was somehow surprising because the rationale to use a given chemotherapy is that it will target preferentially the malignant cells. Nevertheless, the important information drawn from Fig 2B is that TAT-RasGAP_317-326_ does not sensitize PBLs to etoposide-, vincristine- and doxorubicin-mediated death. The peptide however did sometimes sensitize PBLs to 4-HC (Fig 2B), suggesting that TAT-RasGAP_317-326_ can affect the viability of normal cells in some treatment combinations.

### TAT-RasGAP_317-326_ can potentiate genotoxin-induced cell death in Ewing sarcoma

**Fig 3:**
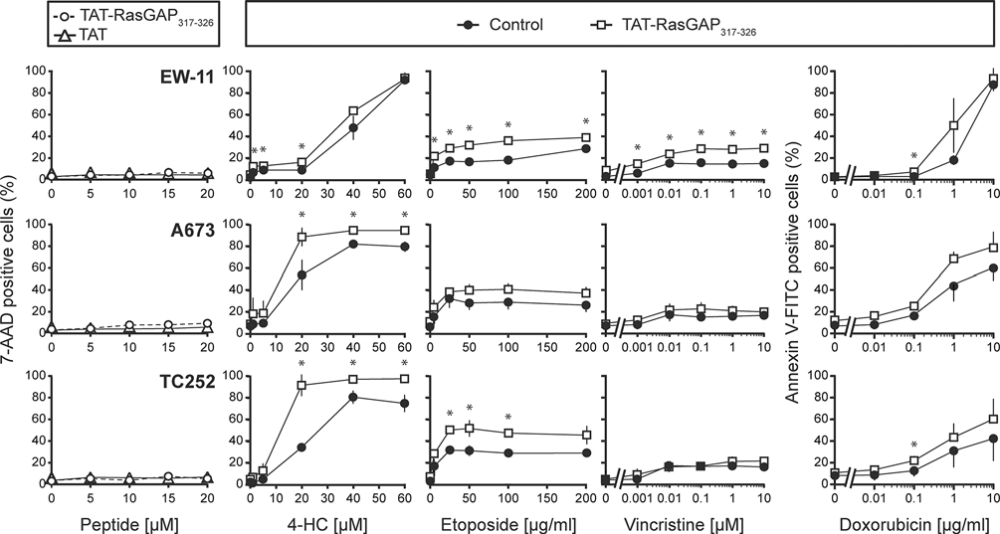
TAT-RasGAP_317-326_ potentiates genotoxin-induced cell death in Ewing sarcoma. Three Ewing sarcoma cell lines (EW-11, A673 and TC252) were seeded in 6-well plates and after 24 hours were treated with 4-HC, etoposide, vincristine, or doxorubicin at the indicated concentrations in the presence or in the absence of 10 μM TAT-RasGAP_317-326_. One day later, cell death was evaluated. 4-HC, 4-hydroperoxycyclophosphamide. * p<0.05 t-test after Bonferroni correction.

To extend the investigation on TAT-RasGAP_317-326_ in non-leukemia childhood cancer, the efficacy of TAT-RasGAP_317-326_ was also tested in Ewing sarcoma cells. The results show that the RasGAP-derived peptide is also able to sensitize these cells to various genotoxins, although to a lower extent than in leukemias (Fig 3). In some cases, no sensitization was observed (e.g. when vincristine was used in the A673 and TC252 cell lines). Here again, TAT-RasGAP_317-326_ alone did not display any toxicity toward the Ewing sarcoma cells (Fig 3).

### The NB1 neuroblastoma derived-cell lines are directly killed by TAT-RasGAP_317-326_

**Fig 4:**
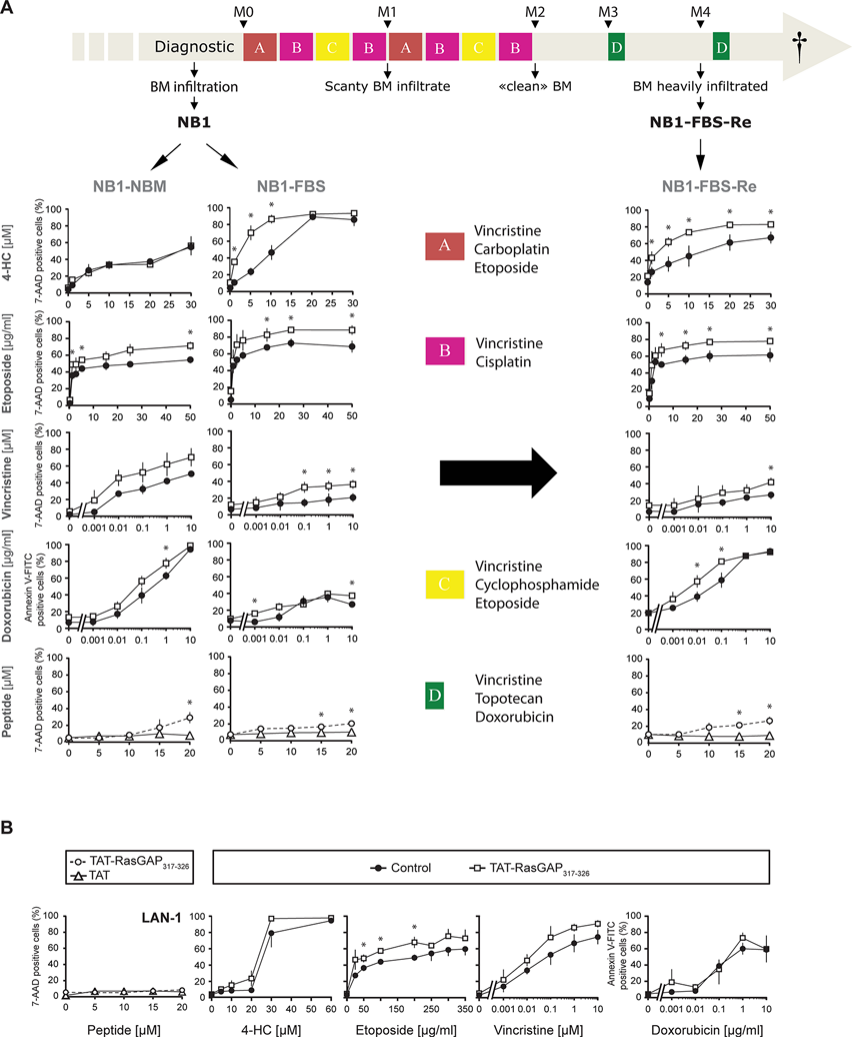
TAT-RasGAP_317-326_ sensitizes neuroblastoma cells to chemotherapy and displays a direct killing effect on NB1-derived cell lines. **A.** The light grey thick arrow on top of the figure represents the treatment regimen administrated to the patient from whom the three NB1-derived cell lines were established. The NB1 cells are derived from primary high risk neuroblastoma bone marrow samples. NB1-NBM and NB1-FBS were established at initial diagnosis and cultured in NBM and DMEM media, respectively. NB1-NBM-Re was established at a subsequent relapse and cultured in DMEM. The three NB1-derived cell lines were treated as described in Fig 3. **B.** The LAN-1 cell line is derived from a high risk neuroblastoma. LAN-1 cells were treated as described in Fig 3. M, month; BM, bone marrow; 4-HC, 4-hydroperoxycyclophosphamide. * p<0.05 t-test after Bonferroni correction.

Acquired chemoresistance is an important cause of failure of treatment of neuroblastoma. To assess the effect of TAT-RasGAP_317-326_ in cells that were exposed to high doses of chemotherapies and therefore that potentially have acquired resistance to these cytotoxic agents, we selected primary tumor cells of a single patient at different stages of the disease. The NB1 cells are high risk stage 4 primary neuroblastoma cells derived from bone marrow samples that were established at initial diagnosis (NB1-NBM and NB1-FBS) and at subsequent relapse after multi-agent chemotherapy (NB1-FBS-Re) (Fig 4A). NB1-FBS and NB1-FBS-Re cell lines were cultured in 10% fetal bovine serum (FBS)-containing DMEM medium, while NB1-NBM cells were established in neural basic medium (NBM). NBM is a stem cell permissive serum-free, bFGF-, EGF- and B27-supplemented medium to support the growth of neural crest cells, the tissue of origin of neuroblastoma [38, 39].

Fig 4A shows that the NB1-NBM and NB1-FBS cell lines do not display the same sensitivity toward the tested chemotherapies. Moreover, the efficacy of TAT-RasGAP_317-326_ to improve the killing efficiency of 4-HC varies between these two cell lines: the NB1-FBS cells were found to be very sensitive to the sensitization effect of the peptide, while the NB1-NBM cells were not affected by the presence of TAT-RasGAP_317-326_. As the only difference between the NB1-NBM and NB1-FBS cell lines is the medium in which they are cultured, one could hypothesize that the metabolism of the NB1 cells varies from one culture condition to the other and that this differentially modulates the sensitivity of the tumor cells to TAT-RasGAP_317-326_.

To investigate whether the sensitization effect of TAT-RasGAP_317-326_ was still efficient on relapsed neuroblastoma cells, we compared its efficacy between the NB1-FBS cells and the NB1-FBS-Re cells. Both cell lines are cultured in the same culture medium. The treatment regimen that was used to treat the patient is illustrated in Fig 4A. The patient, the relapsed cells of which were derived, was exposed to the four drugs (4-HC, etoposide, vincristine and doxorubicin) tested in this study. However, except for the 4-HC-treated conditions where we can observe a slight resistance to this drug in the NB1-FBS-Re cells, the relapsed cells are not more resistant to etoposide, vincristine and doxorubicin than the cells derived from initial diagnosis (Fig 4A). On the contrary, NB1-FBS-Re cells show an increased sensitivity to doxorubicin compared to the NB1-FBS cells. Nevertheless, TAT-RasGAP_317-326_ sensitizes the three primary NB1-derived cell lines, although to a different level of efficacy according to the cell line and the tested chemotherapy (Fig 4A).

In most cases, TAT-RasGAP_317-326_ does not induce tumor cell death by itself. However, we found out that the three NB1 derived-cell lines can be directly killed by the RasGAP-derived peptide (Fig 4A). The dose needed to induce cell death (20 μM) is nevertheless higher than the dose used to sensitize the cells to genotoxins (10 μM).

To complete the investigation of the effect of TAT-RasGAP_317-326_ in neuroblastoma tumor type, we also tested the peptide on LAN-1, another neuroblastoma cell line derived from an aggressive stage 4 neuroblastoma. Fig 4B shows that the RasGAP-derived peptide displays a slight sensitization effect toward this cell line. Unlike the NB1 derived-cell lines, TAT-RasGAP_317-326_ alone was not able to kill LAN-1 cells (Fig 4B), meaning that TAT-RasGAP_317-326_-induced cell death is not a specificity shared by all neuroblastomas.

### The sensitizing activity of TAT-RasGAP_317-326_ is carried by the RasGAP-derived sequence

**Fig 5:**
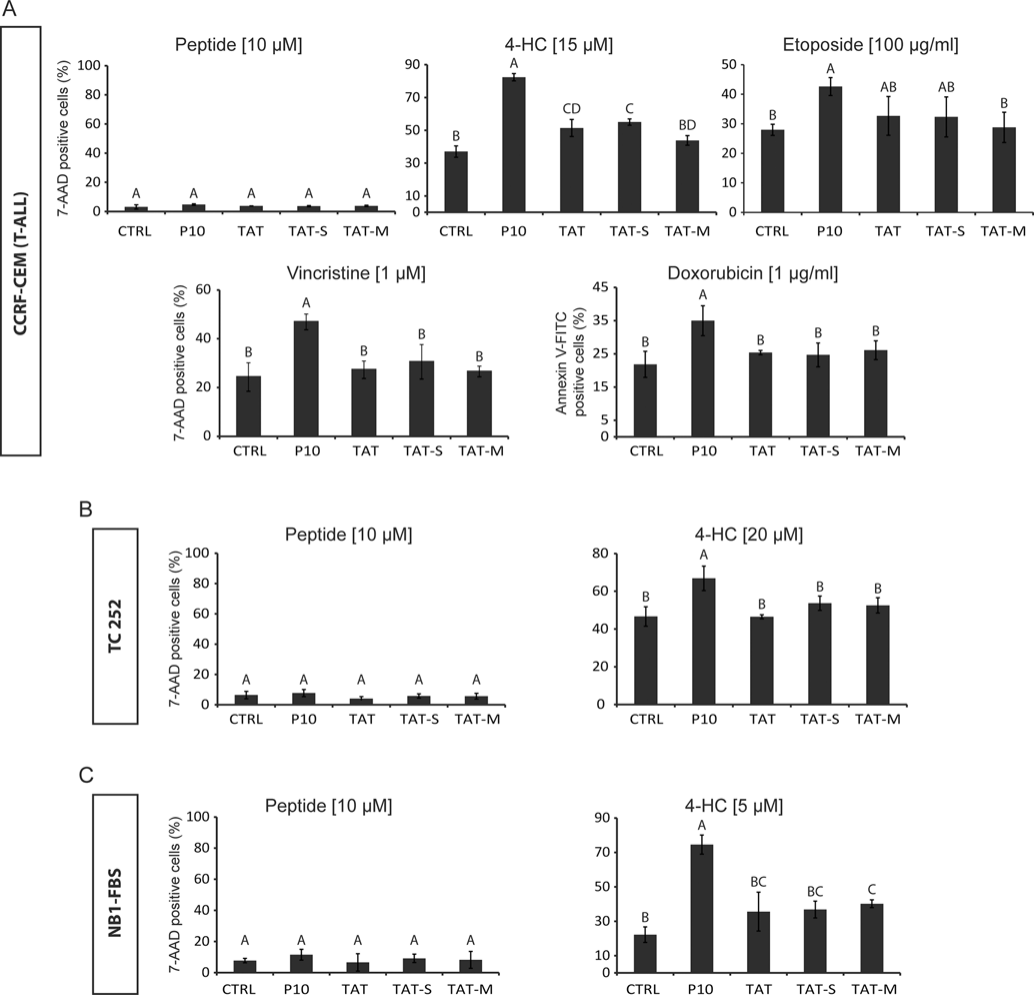
The RasGAP moiety carries the tumor sensitizing activity of TAT-RasGAP_317-326_. **A.** CCRF-CEM cells were seeded in 6-well plates and directly treated with 10 μM TAT-RasGAP_317-326_, TAT, TAT-Scrambled or TAT-Mutated in the absence or in the presence of 4-HC, etoposide, vincristine or doxorubicin at the indicated concentrations. After 24 hours of drug incubation, 7-AAD or Annexin V-FITC staining was performed to evaluate cell death. **B-C.** The TC252 (B) and NB1-FBS (C) cell lines were treated similarly but in combination with 4-HC only. P10, TAT-RasGAP_317-326_; TAT-S, TAT-Scrambled; TAT-M, TAT-Mutated; 4-HC, 4-hydroperoxycyclophosphamide. Means with the same letter are not significantly different.

To assess the specificity of the chemo-sensitizing activity of TAT-RasGAP_317-326_, three different control peptides were tested: a peptide composed of the TAT sequence only (TAT), a peptide in which the RasGAP sequence was scrambled (TAT-Scrambled), and a peptide in which the first tryptophan of the RasGAP sequence was substituted into an alanine residue (TAT-Mutated). This tryptophan was recently shown to be essential for the sensitizing activity of TAT-RasGAP_317-326_ in adult tumors [16]. The effect of TAT-RasGAP_317-326_ and the three control peptides was tested in CCRF-CEM cells in combination with various anti-cancer drugs at doses that allowed the greatest sensitization activity to be detected. CCRF-CEM cells were used because this leukemia cell line was the one most efficiently sensitized by the RasGAP-derived peptide. Fig 5A shows that all three control peptides had a tendency to slightly favor the death of CCRF-CEM cells when combined with the different anti-cancer drugs but in most cases this did not reach statistical significance. In contrast, TAT-RasGAP_317-326_ markedly, and always significantly, sensitized these cells to the drugs. The slight sensitization effect of the control peptides is most likely due to the cell penetrating activity of the TAT moiety, which has the potential to negatively affect cellular homeostasis [40]. Similar results were obtained when the TC252 Ewing sarcoma cell line and the NB1-FBS neuroblastoma cell line were used: TAT-RasGAP_317-326_ significantly increased their sensitivity to 4-HC, while the three control peptides did not, or only minimally (Fig 5B and 5C). These data indicate that the tumor sensitizing activity of TAT-RasGAP_317-326_ only marginally relies on its ability to penetrate cells via the TAT cell-permeable sequence. Therefore, it can be concluded that the sensitizing activity of TAT-RasGAP_317-326_ is mainly carried by the RasGAP-derived sequence.

## Discussion

The currently applied therapies to treat childhood solid cancers, such as neuroblastoma and Ewing sarcoma, need to be more efficient and specific to improve clinical outcome. Leukemia has usually a better 5-year survival rate than pediatric solid tumors, but long-term effects of the therapy remain an important cause of morbidity and mortality. Furthermore, acquired resistance to the conventional treatments is responsible for relapses and poor outcome. Accordingly, finding less toxic and more efficient therapeutic agents to treat pediatric cancers is a necessity to decrease drug-induced deleterious side effects and the associated mortality [3].

In the present study, we assessed the effect of TAT-RasGAP_317-326_ in several childhood cancer cell lines. This RasGAP-derived peptide was already known to sensitize adult tumor cells *in vitro* and *in vivo* to various anti-cancer therapies [12]. Our results show that TAT-RasGAP_317-326_ significantly sensitizes childhood cancer cells in the majority of the tested conditions. However, in some cases, tumor cells did not, or minimally, respond to the peptide. The pediatric tumor-sensitizing effect illustrated in this study was reached using half of the peptide concentration used previously to sensitize adult cancer cells (10 μM TAT-RasGAP_317-326_ in this study instead of 20 μM in the paper of Michod et al. [12]). Possibly, the dose of TAT-RasGAP_317-326_ could be doubled to increase the sensitizing efficacy of the peptide in the conditions where it was less efficient. Since primary tumor cells represent a more clinically relevant condition, we also tested the peptide in primary neuroblastoma cells derived from bone marrow samples from a single patient (the three NB1-derived cell lines showed in Fig 4). The results show that the RasGAP-derived peptide sensitizes primary tumor cells, suggesting that it could exert its anti-cancer activity on tumors in an *in vivo* context.

The mechanism of action of TAT-RasGAP_317-326_ remains to be precisely determined [18–20]. We initially assumed that working with a large panel of cell lines and cytotoxic agents would allow us to draw a pattern that could be used to predict which type of childhood tumors display sensitivity to TAT-RasGAP_317-326_ in response to a given drug. Such information would be helpful to decipher the mode of action of TAT-RasGAP_317-326_. For this purpose, we analyzed how the half maximal inhibitory concentration (IC_50_) for each chemotherapeutic agent was modified by the presence of TAT-RasGAP_317-326_ for a given cell line (Table 2). The RasGAP-derived peptide decreased the IC_50_ for most genotoxins in most cell lines. However, no specific pattern based on tumor origin or drug type could be deduced from the data presented in Table 2. We also looked whether there were some common specific mutations in well-known oncogenes or tumor suppressor genes (e.g. HRAS, KRAS, NRAS, HDM2, N-MYC, C-MYC, TAL1, FLI-1, MLL, p53 and Rb1) in the analyzed cell lines. Again, no association could be found between the presence of specific mutations in a cancer cell line and its ability to be sensitized by the RasGAP-derived peptide.

**Table 2:** Sensitizing effect of the TAT-RasGAP_317-326_ toward childhood tumors.

****Cell line****	****Tumor type****	****Chemotherapy****	****IC****_50_ ****(without peptide)****	****IC****_50_ ****(with 10**** μ****M TAT-RasGAP****_317-326_ ****)****	****Effect of TAT-RasGAP****_317-326_ ****on IC****_50_
THP-1	AML	4-HC [μM]	38	25	Decrease
		Etoposide [μg/ml]	>20	10	Decrease
		Vincristine [μM]	>10	>10	-
		Doxorubicin [μg/ml]	0.3	0.3	No change
M-07e	AML	4-HC [μM]	20	11	Decrease
		Etoposide [μg/ml]	>150	150	Decrease
		Vincristine [μM]	>10	1	Decrease
		Doxorubicin [μg/ml]	2	2	No change
CEM	T-ALL	4-HC [μM]	25	12.5	Decrease
		Etoposide [μg/ml]	>100	25	Decrease
		Vincristine [μM]	>10	>10	-
		Doxorubicin [μg/ml]	>10	>10	-
EW-11	Ewing sarcoma	4-HC [μM]	40	35	Decrease
		Etoposide [μg/ml]	>200	>200	-
		Vincristine [μM]	>10	>10	-
		Doxorubicin [μg/ml]	3	1	Decrease
A673	Ewing sarcoma	4-HC [μM]	20	10	Decrease
		Etoposide [μg/ml]	>200	>200	-
		Vincristine [μM]	>10	>10	-
		Doxorubicin [μg/ml]	2	0.4	Decrease
TC252	Ewing sarcoma	4-HC [μM]	27	10	Decrease
		Etoposide [μg/ml]	>200	25	Decrease
		Vincristine [μM]	>10	>10	-
		Doxorubicin [μg/ml]	>10	3	Decrease
NB1-NBM	Neuroblastoma	4-HC [μM]	27	27	No change
		Etoposide [μg/ml]	25	1	Decrease
		Vincristine [μM]	10	0.04	Decrease
		Doxorubicin [μg/ml]	0.3	0.07	Decrease
NB1-FBS	Neuroblastoma	4-HC [μM]	10	3	Decrease
		Etoposide [μg/ml]	2.5	1	Decrease
		Vincristine [μM]	>10	>10	-
		Doxorubicin [μg/ml]	>10	>10	-
NB1-FBS-Re	Neuroblastoma	4-HC [μM]	15	2.5	Decrease
		Etoposide [μg/ml]	2.5	1	Decrease
		Vincristine [μM]	>10	>10	-
		Doxorubicin [μg/ml]	0.05	0.004	Decrease
LAN-1	Neuroblastoma	4-HC [μM]	26	24	Decrease
		Etoposide [μg/ml]	200	50	Decrease
		Vincristine [μM]	0.1	0.02	Decrease
		Doxorubicin [μg/ml]	0.3	0.3	No change

This table summarizes the effect of TAT-RasGAP_317-326_ in combination with 4-HC, etoposide, vincristine or doxorubicin on the IC_50_ of the cell lines studied in this paper. IC_50_ were calculated for each chemotherapeutic agent in the absence or in the presence of 10 μM TAT-RasGAP_317-326_. AML, acute myeloid leukemia; T-ALL, T-acute lymphoblastic leukemia; 4-HC, 4-hydroperoxycyclophophamide.

An interesting aspect of this research is that the sensitization effect of TAT-RasGAP_317-326_ can be differentially modulated by the cell culture conditions. This feature is illustrated by the fact that the NB1-FBS cells, but not the NB1-NBM cells, are sensitive to the effect of the peptide. These two cell lines are derived from the same neuroblastoma tumor at initial diagnosis (see Fig 4) but they were cultured in different media. These two cell lines appear to have the same genomic alterations. Indeed, they possess identical array-CGH profiles with the same typical neuroblastoma-associated segmental chromosomic alterations (unpublished data). So if these two cell lines have identical array-CGH profiles, what could explain their different sensitivity to TAT-RasGAP_317-326_? One possibility is that the medium in which they are grown modulate their metabolism differentially. This might in turn affect their sensitivity to anti-cancer treatments. There are indeed accumulating evidence that the metabolic state of cancer cells play an important role in the way they respond to anti-cancer drugs [41]. Another hypothesis is that different cell populations are selected upon passages in different medium. Unlike the FBS-containing DMEM medium, the NBM medium blocks cell differentiation. Consequently, the NB1-FBS cells are potentially more differentiated than the NB1-NBM cells. The state of differentiation of the cells could explain their different behavior in response to TAT-RasGAP_317-326_. Alternatively, passage in different medium may induce differential epigenetic modifications or mutations that impact on the sensitivities of the NB1-FBS and NB1-NBM cell lines toward the RasGAP-derived peptide.

We have previously shown that the genotoxin sensitivity of non-tumor cells is not affected by TAT-RasGAP_317-326_ [12]. Moreover, doses that induce tumor sensitization *in vivo* do not exert any detectable toxic side effects [13]. In the present study, we tested the effect of the peptide in isolated lymphocytes from blood of healthy patients. We confirmed the non-toxic effect of the RasGAP-derived peptide in these cells. The peptide was also unable to sensitize the isolated lymphocytes to etoposide, vincristine or doxorubicin. However, TAT-RasGAP_317-326_ was found sometimes to sensitize non-malignant lymphocytes to 4-HC-induced death. This finding is clinically relevant because one of the most frequent dose-limiting side effect of chemotherapy is hematological toxicity [42]. Consequently, if TAT-RasGAP_317-326_ increases genotoxin-induced side effects toward non-tumor cells, it would lose its attractivity as an anti-cancer candidate to treat childhood tumors. However, cyclophosphamide (and consequently its active form 4-HC) possesses hematologic toxic effects. This toxicity depends of the cellular expression level of aldehyde dehydrogenase (ALDH), an enzyme responsible for cyclophosphamide detoxification. The expression level of ALDH is very low in mature hematopoietic cells such as lymphocytes, while in hematopoietic stem cells, ALDH is expressed at high levels. Thus, the later are relatively resistant to cyclophosphamide, whereas this alkylating agent is toxic toward mature lymphocytes [43]. This feature explains why white blood cell counts drop in patients treated with cyclophosphamide [44]. However this drop is transient and a rapid hematologic recovery invariably occurs after cyclophosphamide therapy. Consequently, the observed sensitizing effect of TAT-RasGAP_317-326_ in combination with 4-HC in healthy lymphocytes may not be clinically incompatible, provided that the negative effect of the peptide is limited to mature hematopoietic cells and does not affect hematopoietic stem cells.

Although in most cases TAT-RasGAP_317-326_ by itself does not kill tumor cells, we found out that it induced the cell death of the three NB1-derived cell lines tested here. This newly discovered feature has two consequences. First, it will give us the opportunity to study the pro-death activity of the peptide alone. This possibly may facilitate deciphering its mode of action by, for example, using genome-scale knockout screenings [45, 46]. Second, it indicates that the RasGAP-derived peptide, in its own right, corresponds to a new cytotoxic agent in certain tumors.

In conclusion, our work shows that in most cases TAT-RasGAP_317-326_ sensitizes childhood cancer cells to genotoxins. However, it was not possible to predict the efficacy of this sensitization based on the tumor type and the drug used. Additional investigation is therefore required to increase our understanding on how TAT-RasGAP_317-326_ works and to determine the indications for which it might be useful in the clinic.
